# Bis(acetato-κ^2^ *O*,*O*′)(4,4′-dimethyl-2,2′-bipyridine-κ^2^ *N*,*N*′)copper(II) monohydrate

**DOI:** 10.1107/S1600536812020193

**Published:** 2012-05-16

**Authors:** Aphiwat Kaewthong, Mongkol Sukwattanasinitt, Nongnuj Muangsin

**Affiliations:** a Center for Petroleum, Petrochemicals and Advanced Materials, Department of Chemistry, Faculty of Science, Chulalongkorn University, Bangkok 10330, Thailand; b Research Centre of Bioorganic Chemistry, Department of Chemistry, Faculty of Science, Chulalongkorn University, Bangkok, 10330, Thailand

## Abstract

In the title compound, [Cu(C_2_H_3_O_2_)_2_(C_12_H_12_N_2_)_2_]·H_2_O, the Cu^II^ atom exhibits a distorted octa­hedral coordination geometry, defined by two N atoms from one 4,4′-dimethyl-2,2′-bipyridine ligand and four O atoms from two acetate ligands. In the crystal, O—H⋯O hydrogen bonds are observed between the coordinated carboxyl­ate O atoms and the solvent water mol­ecule.

## Related literature

For related structures, see: Willett *et al.* (2001 ▶); Amani *et al.* (2009 ▶); Hojjat Kashani *et al.* (2008 ▶); Alizadeh *et al.* (2009 ▶, 2010 ▶). For standard bond lengths, see: Allen *et al.* (1987 ▶).
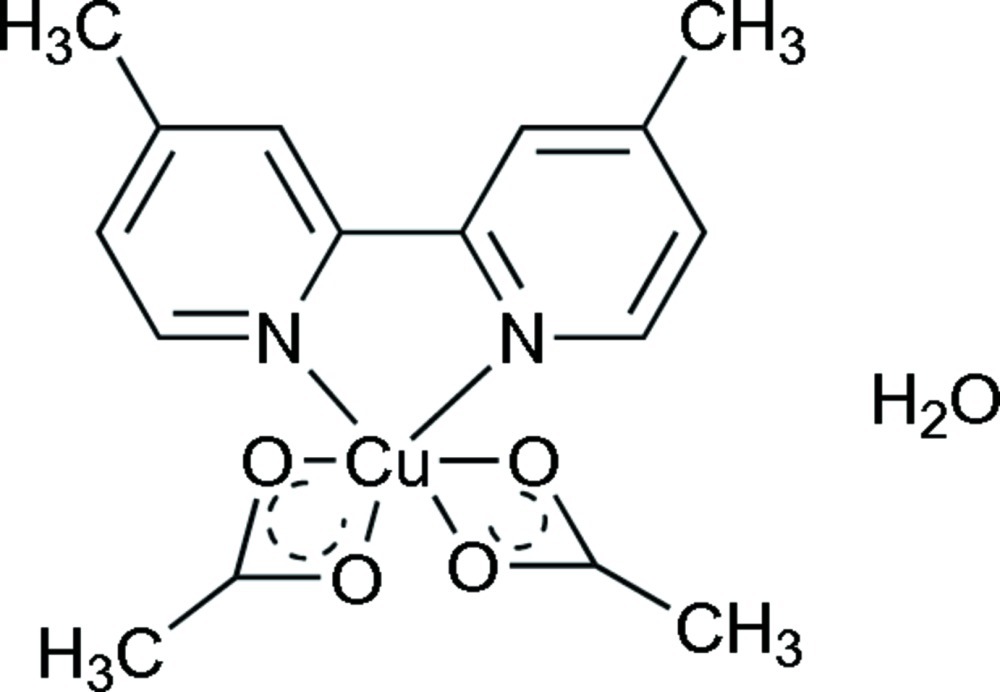


## Experimental

### 

#### Crystal data

[Cu(C_2_H_3_O_2_)_2_(C_12_H_12_N_2_)_2_]·H_2_O
*M* *_r_* = 383.88
Orthorhombic, 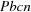

*a* = 22.0667 (8) Å
*b* = 9.0192 (3) Å
*c* = 17.4088 (6) Å
*V* = 3464.8 (2) Å^3^
*Z* = 8
Mo *K*α radiation
μ = 1.29 mm^−1^
*T* = 296 K
0.48 × 0.43 × 0.26 mm

#### Data collection

Bruker SMART APEXII CCD area-detector diffractometer
Absorption correction: multi-scan (*SADABS*; Bruker, 2008 ▶) *T*_min_ = 0.544, *T*_max_ = 0.715
18193 measured reflections
4559 independent reflections
2835 reflections with *I* > 2σ(*I*)
*R*_int_ = 0.091

#### Refinement

*R*[*F* ^2^ > 2σ(*F* ^2^)] = 0.058
*wR*(*F* ^2^) = 0.172
*S* = 1.00
4559 reflections
229 parameters
H atoms treated by a mixture of independent and constrained refinement
Δρ_max_ = 0.46 e Å^−3^
Δρ_min_ = −0.87 e Å^−3^

### 

Data collection: *APEX2* (Bruker, 2008 ▶); cell refinement: *SAINT* (Bruker, 2008 ▶); data reduction: *SAINT*; program(s) used to solve structure: *SHELXS97* (Sheldrick, 2008 ▶); program(s) used to refine structure: *SHELXL97* (Sheldrick, 2008 ▶); molecular graphics: *ORTEP-3* (Farrugia, 1997 ▶); software used to prepare material for publication: *publCIF* (Westrip, 2010 ▶).

## Supplementary Material

Crystal structure: contains datablock(s) global, I. DOI: 10.1107/S1600536812020193/jj2130sup1.cif

Structure factors: contains datablock(s) I. DOI: 10.1107/S1600536812020193/jj2130Isup2.hkl

Additional supplementary materials:  crystallographic information; 3D view; checkCIF report

## Figures and Tables

**Table 1:** Hydrogen-bond geometry (Å, °)

*D*—H⋯*A*	*D*—H	H⋯*A*	*D*⋯*A*	*D*—H⋯*A*
O1*S*—H1*S*⋯O4^i^	0.80 (6)	2.12 (6)	2.878 (4)	158 (5)
O1*S*—H2*S*⋯O1^ii^	0.91 (7)	2.19 (7)	2.876 (4)	132 (6)

Symmetry codes: (i) 
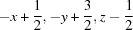
; (ii) 
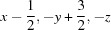
.

**Fig. 1.**
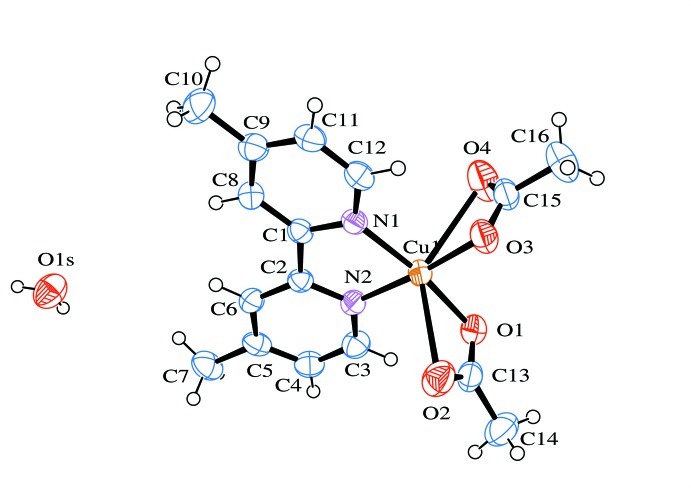
Molecular structure of the title compound showing the atom labeling scheme of the asymmetric unit and 50% probability displacement ellipsoids.

## supplementary crystallographic information

### Comment

The transition metal complexes of 4,4'-dimethyl-2,2'-bipyridine (4,4'-dmbipy) with various secondary types of ligands have been reported, for example, copper with bromide (Willett *et al.*, 2001), iron with chloride (Amani *et al.*, 2009), platinum with chloride (Hojjat Kashani *et al.*, 2008), zinc with bromo (Alizadeh *et al.*, 2010) and zinc with iodide (Alizadeh *et al.*, 2009). Here, we report the title compound, [Cu(CH_3_-bpy)(OAc)_2_].H_2_O, (I), a new copper complex with acetate ligands.

The asymmetric unit of the title compound, (I), is comprised of one water solvent molecule and a [Cu(CH_3_-bpy)(OAc)_2_] complex with a distorted octahedral arrangement around the mononuclear copper (II) group as evidenced by bond angles of N1—Cu1—N2[80.46 (10)^°^], N1—Cu1—O3[94.27 (9)^°^], O1—Cu1—O3[92.91 (10)^°^], O1—Cu1—N2[92.41 (9)^°^]. The six-coordinate geometry around the Cu (II) group is defined by two N atoms from one 4,4'-dimethyl-2,2'-bipyridine ligand and four O atoms from two acetate ligands. Bond lengths and angles are within normal ranges (Allen *et al.*, 1987). In the crystal structure, intermolecular O—H···O hydrogen bonds between the coordinated water molecules and the carboxylate O atoms may help stabilize the structure.

### Experimental

A solution of 4,4'-dimethyl-2,2'-bipyridine (17 mg, 0.1 mmol) and Cu(OAc)_2_.H_2_O (20 mg, 0.1 mmol) in ethanol (10 ml) was stirred and refluxed for 2 h. The solution was placed for slow evaporation at room temperature, and after two weeks X-ray quality crystals of Cu(CH_3_-bpy)(OAc)_2_ appeared as blue prisms. Yield: 23 mg, 60%.

### Refinement

H1S and H2S were located by a difference map and refined isotropically. All the remaiing H atoms were included in calculated positions, with C—H lengths fixed at 0.96 Å (CH_3_) or 0.93 Å (CH). The isotropic displacement parameters for these atoms were set to 1.2 (CH) or 1.5 (CH_3_) times *U*_eq_ of the parent atom.

### Crystal data

**Table:**

[Cu(C_2_H_3_O_2_)_2_(C_12_H_12_N_2_)_2_]·H_2_O	*F*(000) = 1592
*M**_r_* = 383.88	*D*_x_ = 1.472 Mg m^−^^3^
Orthorhombic, *P**b**c**n*	Mo *K*α radiation, λ = 0.71073 Å
Hall symbol: -P 2n 2ab	Cell parameters from 4435 reflections
*a* = 22.0667 (8) Å	θ = 2.3–27.3°
*b* = 9.0192 (3) Å	µ = 1.29 mm^−^^1^
*c* = 17.4088 (6) Å	*T* = 296 K
*V* = 3464.8 (2) Å^3^	Prism, blue
*Z* = 8	0.48 × 0.43 × 0.26 mm

### Data collection

**Table:**

Bruker SMART APEXII CCD area-detector diffractometer	4559 independent reflections
Radiation source: sealed X-ray tube	2835 reflections with *I* > 2σ(*I*)
Graphite monochromator	*R*_int_ = 0.091
Detector resolution: 8.33 pixels mm^-1^	θ_max_ = 29.0°, θ_min_ = 2.3°
φ and ω scans	*h* = −29→27
Absorption correction: multi-scan (*SADABS*; Bruker, 2008)	*k* = −12→12
*T*_min_ = 0.544, *T*_max_ = 0.715	*l* = −21→23
18193 measured reflections	

### Refinement

**Table:**

Refinement on *F*^2^	Primary atom site location: structure-invariant direct methods
Least-squares matrix: full	Secondary atom site location: difference Fourier map
*R*[*F*^2^ > 2σ(*F*^2^)] = 0.058	Hydrogen site location: inferred from neighbouring sites
*wR*(*F*^2^) = 0.172	H atoms treated by a mixture of independent and constrained refinement
*S* = 1.00	*w* = 1/[σ^2^(*F*_o_^2^) + (0.0896*P*)^2^] where *P* = (*F*_o_^2^ + 2*F*_c_^2^)/3
4559 reflections	(Δ/σ)_max_ = 0.014
229 parameters	Δρ_max_ = 0.46 e Å^−^^3^
0 restraints	Δρ_min_ = −0.87 e Å^−^^3^

### Special details

**Table:**

Experimental. IR [KBr, cm^-1^]: 3426, 3100, 1608, 1444, 1118, 770, 621. HRMS (ESI): m/*z* 389 [*M*+Na]^+^.
Refinement. Refinement of *F*^2^ against ALL reflections. The weighted *R*-factor *wR* and goodness of fit *S* are based on *F*^2^, conventional *R*-factors *R* are based on *F*, with *F* set to zero for negative *F*^2^. The threshold expression of *F*^2^ > σ(*F*^2^) is used only for calculating *R*-factors(gt) *etc*. and is not relevant to the choice of reflections for refinement. *R*-factors based on *F*^2^ are statistically about twice as large as those based on *F*, and *R*- factors based on ALL data will be even larger.

### Fractional atomic coordinates and isotropic or equivalent isotropic displacement parameters (Å^2^)

**Table:**

	*x*	*y*	*z*	*U*_iso_*/*U*_eq_	
C1	0.23138 (13)	0.3008 (3)	0.11076 (14)	0.0337 (6)	
C2	0.25879 (12)	0.3929 (3)	0.04944 (14)	0.0339 (6)	
C3	0.34865 (14)	0.4750 (3)	−0.00435 (16)	0.0448 (7)	
H3	0.3908	0.4767	−0.0051	0.054*	
C4	0.31721 (15)	0.5568 (3)	−0.05769 (16)	0.0456 (7)	
H4	0.3384	0.6127	−0.0936	0.055*	
C5	0.25498 (15)	0.5573 (3)	−0.05882 (14)	0.0412 (6)	
C6	0.22514 (13)	0.4722 (3)	−0.00311 (15)	0.0379 (6)	
H6	0.183	0.4693	−0.0016	0.046*	
C7	0.21977 (17)	0.6439 (4)	−0.11742 (17)	0.0546 (9)	
H7A	0.2403	0.7355	−0.1279	0.082*	
H7B	0.18	0.6644	−0.0979	0.082*	
H7C	0.2166	0.5871	−0.1639	0.082*	
C8	0.17017 (14)	0.2973 (4)	0.12849 (15)	0.0404 (6)	
H8	0.1434	0.3574	0.1015	0.049*	
C9	0.14843 (14)	0.2055 (4)	0.18599 (17)	0.0428 (7)	
C10	0.08269 (16)	0.2018 (5)	0.2063 (2)	0.0665 (10)	
H10A	0.0642	0.2941	0.1921	0.1*	
H10B	0.0783	0.1867	0.2606	0.1*	
H10C	0.0633	0.1221	0.1792	0.1*	
C11	0.19084 (14)	0.1170 (3)	0.22375 (16)	0.0421 (7)	
H11	0.1782	0.0509	0.2615	0.051*	
C12	0.25113 (14)	0.1271 (3)	0.20532 (16)	0.0422 (6)	
H12	0.2787	0.0689	0.2323	0.051*	
C13	0.44518 (14)	0.1966 (4)	0.02471 (18)	0.0472 (7)	
C14	0.50052 (17)	0.2074 (5)	−0.0242 (2)	0.0669 (10)	
H14A	0.5124	0.1101	−0.0407	0.1*	
H14B	0.5328	0.2513	0.005	0.1*	
H14C	0.492	0.2679	−0.0683	0.1*	
C15	0.40175 (14)	0.1801 (4)	0.25884 (17)	0.0465 (7)	
C16	0.4320 (2)	0.0955 (4)	0.3226 (2)	0.0695 (11)	
H16A	0.4746	0.1167	0.3226	0.104*	
H16B	0.4258	−0.0088	0.3151	0.104*	
H16C	0.4148	0.1248	0.371	0.104*	
N1	0.27219 (11)	0.2172 (3)	0.15003 (14)	0.0353 (5)	
N2	0.32009 (11)	0.3924 (2)	0.04910 (12)	0.0369 (5)	
O1	0.44010 (10)	0.2880 (3)	0.08035 (12)	0.0499 (5)	
O1S	0.03896 (17)	1.0232 (4)	−0.12525 (17)	0.0744 (8)	
H1S	0.062 (3)	1.080 (6)	−0.145 (3)	0.11 (2)*	
H2S	0.003 (3)	1.064 (8)	−0.140 (4)	0.17 (3)*	
O2	0.40469 (11)	0.1061 (3)	0.00996 (14)	0.0628 (6)	
O3	0.38982 (10)	0.1081 (2)	0.19769 (12)	0.0512 (5)	
O4	0.38908 (14)	0.3131 (3)	0.26600 (15)	0.0721 (8)	
Cu1	0.359174 (16)	0.24915 (3)	0.122075 (19)	0.03621 (15)	

### Atomic displacement parameters (Å^2^)

**Table:**

	*U*^11^	*U*^22^	*U*^33^	*U*^12^	*U*^13^	*U*^23^
C1	0.0374 (15)	0.0331 (13)	0.0306 (12)	0.0006 (11)	−0.0018 (10)	−0.0027 (11)
C2	0.0383 (15)	0.0319 (13)	0.0315 (13)	0.0038 (10)	0.0012 (11)	−0.0039 (10)
C3	0.0423 (16)	0.0437 (16)	0.0482 (17)	−0.0031 (13)	0.0049 (13)	0.0047 (13)
C4	0.0516 (19)	0.0430 (17)	0.0422 (15)	−0.0043 (12)	0.0065 (13)	0.0064 (12)
C5	0.0577 (19)	0.0337 (14)	0.0321 (13)	0.0085 (11)	0.0003 (13)	−0.0005 (11)
C6	0.0361 (15)	0.0401 (15)	0.0376 (14)	0.0067 (11)	0.0016 (11)	−0.0004 (12)
C7	0.072 (2)	0.0497 (19)	0.0425 (17)	0.0115 (16)	−0.0060 (14)	0.0082 (14)
C8	0.0360 (16)	0.0460 (15)	0.0393 (15)	0.0019 (13)	−0.0026 (12)	−0.0010 (12)
C9	0.0431 (17)	0.0462 (15)	0.0392 (16)	−0.0104 (12)	0.0023 (12)	−0.0018 (13)
C10	0.042 (2)	0.088 (3)	0.070 (2)	−0.0099 (18)	0.0070 (17)	0.019 (2)
C11	0.0495 (18)	0.0436 (16)	0.0332 (13)	−0.0080 (13)	0.0008 (12)	0.0018 (12)
C12	0.0500 (18)	0.0403 (15)	0.0362 (14)	0.0007 (12)	−0.0036 (13)	0.0051 (11)
C13	0.0342 (16)	0.0573 (18)	0.0501 (17)	0.0060 (14)	−0.0013 (13)	0.0084 (15)
C14	0.050 (2)	0.079 (2)	0.072 (2)	0.0076 (18)	0.0164 (18)	0.008 (2)
C15	0.0403 (17)	0.056 (2)	0.0436 (16)	0.0002 (14)	−0.0090 (13)	0.0072 (14)
C16	0.077 (3)	0.072 (2)	0.060 (2)	0.0003 (19)	−0.0247 (19)	0.0156 (19)
N1	0.0384 (13)	0.0371 (12)	0.0305 (11)	0.0022 (9)	−0.0003 (10)	0.0011 (9)
N2	0.0348 (12)	0.0389 (12)	0.0371 (12)	0.0010 (9)	0.0012 (9)	0.0013 (10)
O1	0.0354 (12)	0.0657 (13)	0.0487 (13)	0.0004 (10)	−0.0037 (9)	0.0006 (11)
O1S	0.065 (2)	0.0660 (18)	0.092 (2)	0.0099 (15)	0.0102 (16)	0.0145 (16)
O2	0.0445 (13)	0.0694 (16)	0.0743 (16)	−0.0041 (11)	0.0014 (12)	−0.0130 (12)
O3	0.0515 (13)	0.0535 (13)	0.0486 (12)	0.0073 (10)	−0.0124 (10)	0.0020 (10)
O4	0.087 (2)	0.0588 (15)	0.0709 (16)	0.0163 (15)	−0.0301 (14)	−0.0092 (14)
Cu1	0.0334 (2)	0.0398 (3)	0.0354 (2)	0.00240 (13)	−0.00412 (13)	−0.00033 (13)

### Geometric parameters (Å, º)

**Table:**

C1—N1	1.359 (4)	C11—C12	1.372 (4)
C1—C8	1.386 (4)	C11—H11	0.93
C1—C2	1.482 (4)	C12—N1	1.343 (4)
C2—N2	1.353 (3)	C12—H12	0.93
C2—C6	1.378 (4)	C13—O2	1.237 (4)
C3—N2	1.349 (3)	C13—O1	1.277 (4)
C3—C4	1.374 (4)	C13—C14	1.492 (5)
C3—H3	0.93	C14—H14A	0.96
C4—C5	1.373 (5)	C14—H14B	0.96
C4—H4	0.93	C14—H14C	0.96
C5—C6	1.401 (4)	C15—O4	1.238 (4)
C5—C7	1.501 (4)	C15—O3	1.275 (4)
C6—H6	0.93	C15—C16	1.504 (4)
C7—H7A	0.96	C16—H16A	0.96
C7—H7B	0.96	C16—H16B	0.96
C7—H7C	0.96	C16—H16C	0.96
C8—C9	1.385 (4)	N1—Cu1	2.001 (2)
C8—H8	0.93	N2—Cu1	2.007 (2)
C9—C11	1.395 (4)	O1—Cu1	1.959 (2)
C9—C10	1.493 (5)	O1S—H1S	0.80 (6)
C10—H10A	0.96	O1S—H2S	0.91 (7)
C10—H10B	0.96	O3—Cu1	1.952 (2)
C10—H10C	0.96		
			
N1—C1—C8	121.4 (3)	C9—C11—H11	119.9
N1—C1—C2	113.8 (2)	N1—C12—C11	122.9 (3)
C8—C1—C2	124.8 (3)	N1—C12—H12	118.5
N2—C2—C6	122.5 (2)	C11—C12—H12	118.5
N2—C2—C1	114.2 (2)	O2—C13—O1	121.3 (3)
C6—C2—C1	123.3 (3)	O2—C13—C14	121.1 (3)
N2—C3—C4	121.8 (3)	O1—C13—C14	117.5 (3)
N2—C3—H3	119.1	C13—C14—H14A	109.5
C4—C3—H3	119.1	C13—C14—H14B	109.5
C5—C4—C3	121.1 (3)	H14A—C14—H14B	109.5
C5—C4—H4	119.4	C13—C14—H14C	109.5
C3—C4—H4	119.4	H14A—C14—H14C	109.5
C4—C5—C6	117.3 (2)	H14B—C14—H14C	109.5
C4—C5—C7	121.9 (3)	O4—C15—O3	122.1 (3)
C6—C5—C7	120.8 (3)	O4—C15—C16	121.2 (3)
C2—C6—C5	119.4 (3)	O3—C15—C16	116.8 (3)
C2—C6—H6	120.3	C15—C16—H16A	109.5
C5—C6—H6	120.3	C15—C16—H16B	109.5
C5—C7—H7A	109.5	H16A—C16—H16B	109.5
C5—C7—H7B	109.5	C15—C16—H16C	109.5
H7A—C7—H7B	109.5	H16A—C16—H16C	109.5
C5—C7—H7C	109.5	H16B—C16—H16C	109.5
H7A—C7—H7C	109.5	C12—N1—C1	117.9 (3)
H7B—C7—H7C	109.5	C12—N1—Cu1	126.4 (2)
C9—C8—C1	120.8 (3)	C1—N1—Cu1	115.70 (19)
C9—C8—H8	119.6	C3—N2—C2	117.9 (2)
C1—C8—H8	119.6	C3—N2—Cu1	126.3 (2)
C8—C9—C11	116.8 (3)	C2—N2—Cu1	115.43 (16)
C8—C9—C10	121.4 (3)	C13—O1—Cu1	104.23 (19)
C11—C9—C10	121.8 (3)	H1S—O1S—H2S	100 (5)
C9—C10—H10A	109.5	C15—O3—Cu1	107.6 (2)
C9—C10—H10B	109.5	O3—Cu1—O1	92.91 (10)
H10A—C10—H10B	109.5	O3—Cu1—N1	94.27 (9)
C9—C10—H10C	109.5	O1—Cu1—N1	171.94 (9)
H10A—C10—H10C	109.5	O3—Cu1—N2	174.64 (9)
H10B—C10—H10C	109.5	O1—Cu1—N2	92.41 (9)
C12—C11—C9	120.2 (3)	N1—Cu1—N2	80.46 (10)
C12—C11—H11	119.9		
			
N1—C1—C2—N2	−7.7 (3)	C2—C1—N1—Cu1	4.5 (3)
C8—C1—C2—N2	172.0 (3)	C4—C3—N2—C2	−0.6 (4)
N1—C1—C2—C6	172.1 (2)	C4—C3—N2—Cu1	172.2 (2)
C8—C1—C2—C6	−8.2 (4)	C6—C2—N2—C3	1.0 (4)
N2—C3—C4—C5	−0.2 (4)	C1—C2—N2—C3	−179.2 (2)
C3—C4—C5—C6	0.5 (4)	C6—C2—N2—Cu1	−172.5 (2)
C3—C4—C5—C7	−179.0 (3)	C1—C2—N2—Cu1	7.3 (3)
N2—C2—C6—C5	−0.7 (4)	O2—C13—O1—Cu1	−2.6 (4)
C1—C2—C6—C5	179.5 (2)	C14—C13—O1—Cu1	174.6 (3)
C4—C5—C6—C2	−0.1 (4)	O4—C15—O3—Cu1	6.1 (4)
C7—C5—C6—C2	179.4 (3)	C16—C15—O3—Cu1	−174.1 (3)
N1—C1—C8—C9	−1.5 (4)	C15—O3—Cu1—O1	90.9 (2)
C2—C1—C8—C9	178.8 (3)	C15—O3—Cu1—N1	−92.8 (2)
C1—C8—C9—C11	−0.7 (4)	C13—O1—Cu1—O3	92.8 (2)
C1—C8—C9—C10	179.4 (3)	C13—O1—Cu1—N2	−87.8 (2)
C8—C9—C11—C12	2.3 (4)	C12—N1—Cu1—O3	1.4 (2)
C10—C9—C11—C12	−177.8 (3)	C1—N1—Cu1—O3	178.4 (2)
C9—C11—C12—N1	−1.8 (4)	C12—N1—Cu1—N2	−177.6 (2)
C11—C12—N1—C1	−0.4 (4)	C1—N1—Cu1—N2	−0.6 (2)
C11—C12—N1—Cu1	176.5 (2)	C3—N2—Cu1—O1	−0.5 (2)
C8—C1—N1—C12	2.1 (4)	C2—N2—Cu1—O1	172.35 (18)
C2—C1—N1—C12	−178.2 (2)	C3—N2—Cu1—N1	−176.8 (2)
C8—C1—N1—Cu1	−175.2 (2)	C2—N2—Cu1—N1	−3.87 (18)

### Hydrogen-bond geometry (Å, º)

**Table:**

*D*—H···*A*	*D*—H	H···*A*	*D*···*A*	*D*—H···*A*
O1*S*—H1*S*···O4^i^	0.80 (6)	2.12 (6)	2.878 (4)	158 (5)
O1*S*—H2*S*···O1^ii^	0.91 (7)	2.19 (7)	2.876 (4)	132 (6)

Symmetry codes: (i) −*x*+1/2, −*y*+3/2, *z*−1/2; (ii) *x*−1/2, −*y*+3/2, −*z*.
